# TraRECo: a greedy approach based de novo transcriptome assembler with read error correction using consensus matrix

**DOI:** 10.1186/s12864-018-5034-x

**Published:** 2018-09-04

**Authors:** Seokhyun Yoon, Daeseung Kim, Keunsoo Kang, Woong June Park

**Affiliations:** 1 Department of Electronics Eng., College of Engineering, Dankook University, Yongin-si, Korea; 2 Department of Microbiology, College of Natural Sciences, Dankook University, Cheonan-si, Korea; 3 Department of Molecular Biology, College of Natural Sciences, Dankook University, Cheonan-si, Korea

**Keywords:** RNA-Seq, de novo transcriptome assembly, greedy approach, consensus matrix, read error correction

## Abstract

**Background:**

The challenges when developing a good de novo transcriptome assembler include how to deal with read errors and sequence repeats. Almost all de novo assemblers utilize a de Bruijn graph, with which complexity grows linearly with data size while suffering from errors and repeats. Although one can correct the errors by inspecting the topological structure of the graph, this is not an easy task when there are too many branches. Two research directions are to improve either the graph reliability or the path search precision, and in this study, we focused on the former.

**Results:**

We present TraRECo, a greedy approach to de novo assembly employing error-aware graph construction. In the proposed approach, we built contigs by direct read alignment within a distance margin and performed a junction search to construct splicing graphs. While doing so, a contig of length *l* was represented by a 4 × *l* matrix (called a consensus matrix), in which each element was the base count of the aligned reads so far. A representative sequence was obtained by taking the majority in each column of the consensus matrix to be used for further read alignment. Once the splicing graphs had been obtained, we used IsoLasso to find paths with a noticeable read depth. The experiments using real and simulated reads show that the method provided considerable improvement in sensitivity and moderately better performance when comparing sensitivity and precision. This was achieved by the error-aware graph construction using the consensus matrix, with which the reads having errors were made usable for the graph construction (otherwise, they might have been eventually discarded). This improved the quality of the coverage depth information used in the subsequent path search step and finally the reliability of the graph.

**Conclusions:**

De novo assembly is mainly used to explore undiscovered isoforms and must be able to represent as many reads as possible in an efficient way. In this sense, TraRECo provides us with a potential alternative for improving graph reliability even though the computational burden is much higher than the single *k*-mer in the de Bruijn graph approach.

## Background

Low-cost and high-throughput transcriptome profiling (a.k.a. RNA-seq) based on the next generation sequencing technology has ignited the recent development of many software tools for transcriptome assembly to explore novel isoforms, the co-expression of related genes, differentially expressed genes, and so on. Transcriptome assembly for analyzing such RNA-seq data is categorized as either reference-based or de novo. In reference-based assembly methods such as Cufflinks [1], Scripture [2], IsoLasso [3], and Isoinfer [4], raw RNA-seq reads are first mapped to a reference genome using a splice-aware aligner such as TopHat [5] and we use gene-annotation information to explore the expressed transcript/isoforms and their expression level. Meanwhile, in de novo assembly, raw RNA-seq reads are aligned with each other for constructing a graph directly to represent possible splicing patterns through which isoform detection and expression-level estimation can be performed. Although reference-based assembly provides more accurate isoform detection and abundance estimation, it relies highly on the prior knowledge of known genes (i.e. it requires a known genome) and cannot be applied to a species without a reference genome. Due to these reasons, recent research has focused more on de novo assembly and many de novo assemblers have been proposed so far, including Trans-ABySS [6], Trinity [7], Velvet-OASES [8, 9], IDBA-tran [10], SOAPdenovo-Trans [11], Bridger [12], and more recently, BinPacker [13] and Shannon [14].

The challenges when developing a good transcriptome assembler include how to deal with read errors and sequence repeats that frequently occur in eukaryotes. Almost all of the de novo transcriptome assemblers utilize a de Bruijn graph, which is constructed by converting base sequences into *k*-mer sequences and counting the latter. This approach has been widely adopted in most of the de novo transcriptome assemblers since its complexity grows linearly with the read data size. However, a weakness of the de Bruijn graph-based approach is the difficulty in correcting read errors. One way to deal with them is to use pre-error correction algorithms such as Trimmomatic [15] or QuorUM [16] based on the quality score of the reads, but this approach is a little risky as the quality score only tells us the probability. Other pre-error correction algorithms include Coral [17], SEECER [18], and Rcorrector [19], which are based either on multiple alignments or on the probabilistic approach. Another way to handle read errors, especially in de Bruijn graph-based approaches, is to utilize the topological structure of the graph. That is to say, read errors create many branches with relatively low read coverage and one can simply remove these suspicious branches or merge them with the main branch if it is possible to identify the latter. IDBA-tran [10] is another recently proposed approach in which the authors employed an iterative remapping of reads to the de Bruijn graph with increasing *k*-mer values.

Another drawback of the de Bruijn graph-based approach is that it cannot utilize the full connection information of a read. Most of the de Bruijn graph-based de novo assemblers use a *k*-mer of around 30, which is much smaller than a typical read length of 75 or 100 bp. This means that the effective read length is only 30 bp. As a matter of fact, with a small value of *k*, two or more isoforms that have common sequences longer than *k* can be merged into a single (sometimes huge) graph, thus making isoform detection complicated and decreasing the prediction accuracy. To overcome this tradeoff, Schulz et al. [8] proposed combining the splicing graphs obtained with different *k*-mers, which they showed was effective in resolving this problem.

Based on the research into de novo transcriptome assembly so far, there are two main research directions. One is to improve the precision of plausible path search for given splicing graphs, as demonstrated in Bridger [12] and BinPacker [13], and the other is to improve the reliability in the splicing graph construction, for example, by utilizing the multi *k*-mers approach as in OASES [8] and IDBA-tran [10].

In this study, we explored the latter direction based on a greedy approach employing an error-aware construction of the splicing graph. To overcome read errors and sequence repeats, we built splicing graphs by directly aligning reads, which made us able to resort full connection information of a read, even though the running time was much longer than the single *k*-mer in the de Bruijn graph-based approach. As provided in [20], there are two approaches to genome assembly: overlap-layout-consensus (OLC) and the de Bruijn graph approach. The former first gathers the alignment information between all of the pairs of reads to build overlap graphs, bundles the stretches to obtain contigs, and finally aligns the contigs to correct possible errors by taking a consensus at each position of the aligned contigs. The proposed approach is similar to the former but with a clear difference in that aligning the reads and taking the consensus (error correction) are performed simultaneously during the entire course of the graph construction step. In our scheme, a contig of length *l* is represented by a consensus matrix of size 4 x *l* in which each element is the base count of the reads aligned to that contig and a representative sequence of letters corresponding to the majority (the row index with the highest count) for each column is used for further read alignments. Once an overlap is detected for a read within some distance margin, the consensus matrix is updated by increasing those elements corresponding to the letters of the read. Using this error-aware alignment procedure, we could improve the reliability of the splicing graph by making additional reads having errors usable in the graph construction step. In fact, many of the reads with errors are eventually discarded, as they could not be aligned because of errors, resulting in poor splicing graph. Therefore, adding these reads improves the coverage depth information used in the subsequent path search steps. Although two or more isoforms having similar sequences can be merged to a single graph, this is not mostly due to short repeats but to similar sequences as we can set the minimum overlap threshold to be much longer than that used in the de Bruijn graph-based approach. Note that the consensus matrix tracks the alignment records throughout the entire assembly process and, by inspecting each column of the consensus matrix, one can check whether similar sequences have been merged together or not. If any row other than the representative one is conspicuous, then this is an indication of merged sequences.

## Results

To demonstrate the effectiveness of the proposed scheme, we used three data sets: one was simulated reads with the exact set of target references and their expression levels and the other two were real RNA-seq data (human and mouse) from the gene expression omnibus available at (https://www.ncbi.nlm.nih.gov/geo/).

### Using BLASTN

The primary criterion for performance comparison was the number of ‘distinct’ pairs of the reference-candidate transcript for a given minimum target coverage where the coverage was the ratio of the alignment length covered by a candidate (assembled) transcript to the length of the reference transcript. The coverage can be obtained using the BLASTN software [21], which aligns query sequences to a subject sequence database. We used a reference transcript as the query and a candidate as the subject sequence. By running BLASTN, we obtained the following for each query (reference)–subject (candidate) sequence pair: the query sequence length (*L*_*q*_), the subject sequence length (*L*_*s*_), and the alignment length (*L*_*a*_). The coverage is defined simply as the maximum of *L*_*a*_/*L*_*q*_ over all of the candidates. We set the maximum number of target sequence option to 1, which means that we selected the best matching candidate for each reference transcript. Note that even with this option, a candidate transcript can be matched to multiple references. Hence, we looked up the match list (i.e. the output from BLASTN) to select only the best match for a particular reference-candidate pair. The selected pair was counted as a recovered transcript if the ratio of the alignment length to its paired reference was larger than or equal to the target value.

### Sensitivity and precision as the primary criteria

If the exact set of transcripts that reside in a sample is known, one can measure the sensitivity (the percentage of the recovered transcripts in the total number of target references), which provides the simulated read. However, for real reads, we do not know the exact set of targets, so we compare the number of recovered transcripts for the data. In the evaluation of sensitivity, we specifically allow each candidate transcript and reference to have only one match. However, a multiple of references are sometimes matched to one candidate transcript due to artificial gene fusion, and in these cases, one can define wide sense (or extended) sensitivity where we allow multiple transcripts to match with the same candidate for a given target coverage. Another primary performance criterion is precision as a measure of the compactness of an assembly, which is defined as the percentage of true positives among all of the candidate (assembled) transcripts found with a specific assembler. In fact, there exists a trade-off between the two performance criteria (sensitivity and precision) and we need to compare both measures at the same time for specific target coverage, for which we plot sensitivity versus precision (as shown later on).

### The results for the real reads

#### Data sets

First, we performed de novo assemblies with the two real data sets (human and mouse). The human sample was obtained from the National Center for Biotechnology Information (NCBI) website (accession code SRR445718), which was sequenced from embryonic stem cells derived from human preimplantation embryos and is available in Sequence Read Archive (SRA) format from the NCBI. SRR445718 contains approximately 33,000,000 single-end reads, each with a nominal read length of 100. The mouse sample (accession code SRX062280) contains approximately 53,000,000 paired-end reads, each with a nominal read length of 76 bp; it has been used to test de novo assemblers in many studies.

#### Parameter settings and pre/post processing

We compared TraRECo with some popular de novo assemblers: Trinity (version 2.4.0), Velvet (version 1.2.10) + Oases (version 0.2.02), SOAPdenovo-Trans (version 1.01), TransABySS (version 1.5.2), Bridger (version 2014 − 12-01), and BinPacker (version 1.0). For SOAPdenovo-Trans and Trans-ABySS, we set the *k*-mer to 31 and 32, respectively, with which we were able to obtain the best results, and for Trinity, we used 25 (the default value). For Bridger and BinPacker, we used a *k*-mer length of 25 (the default value) for the human sample and 31 for the mouse one (as the authors originally suggested). With Velvet+Oases, we performed a multi-*k* assembly with *k* ranging from 21 to 37 in steps of 4. As is discussed later, TraRECo has several parameters that need to be set: the normalized distance threshold (*D*_*th*_) to 0.03~ 0.06, the overlap threshold (*O*_*th*_) to 52 (human) and 44 (mouse), and the connection threshold (*C*_*th*_) and the junction overlap threshold (*J*_*th*_), both to 24. In the same way as with other assemblers, we discarded those candidates with a length shorter than 200 bp. For preprocessing, we first used Cutadapt [22] to remove any adaptor sequences remaining in the reads, except for Trinity, for which we enabled the Trimmomatic read trimming option instead. Using the trimmed data, we obtained assembled transcript candidates and, finally, BLASTN was used to align each candidate to the reference transcriptome, for which we used the Ensembl transcript for human (hg19) and mouse (mm9), respectively, to finally obtain the number of recovered reference transcripts for the given target coverage.

#### The impact of distance threshold (D_th_) and coverage depth threshold (CD_th_) in TraRECo

**Fig. 1:**
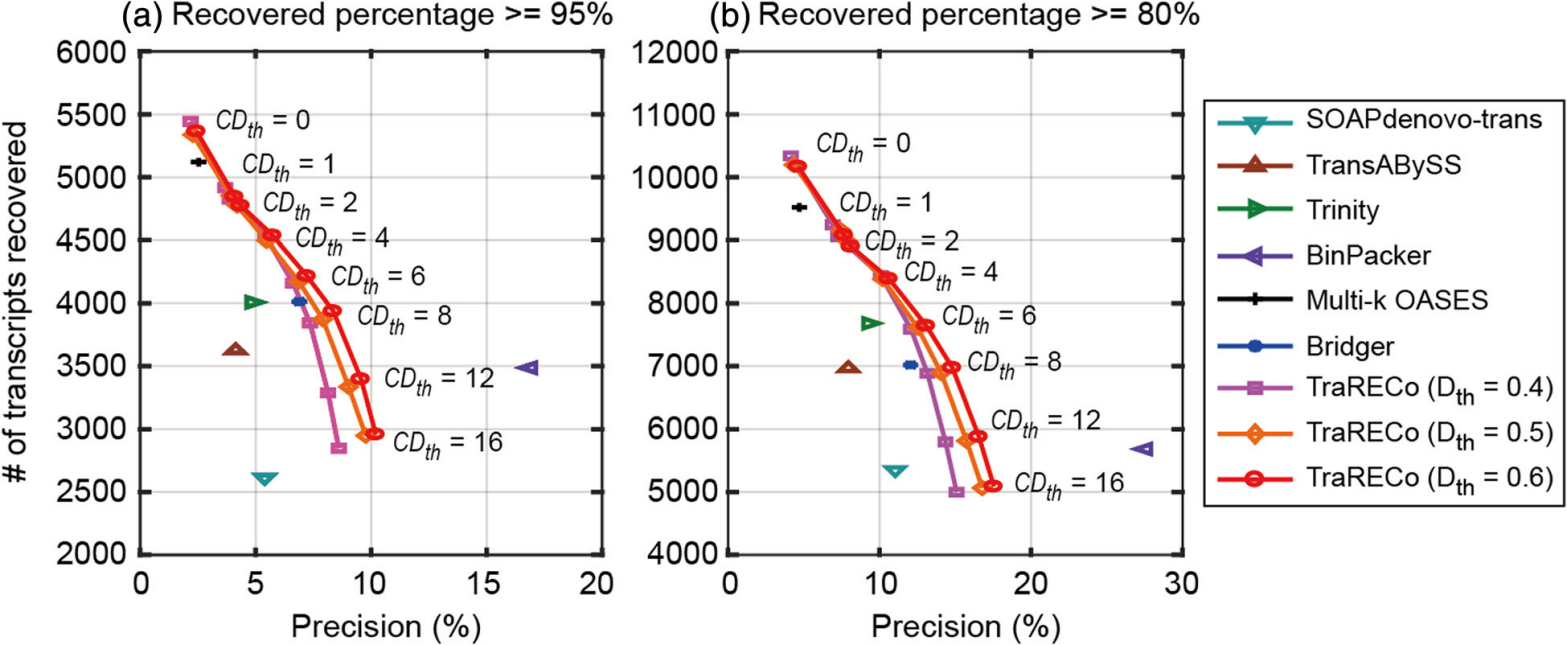
The number of transcripts recovered versus precision for the single-end read data SRR445718 (Human sample). Target coverage = 95% (**a**) and 80% (**b**), respectively

**Fig. 2:**
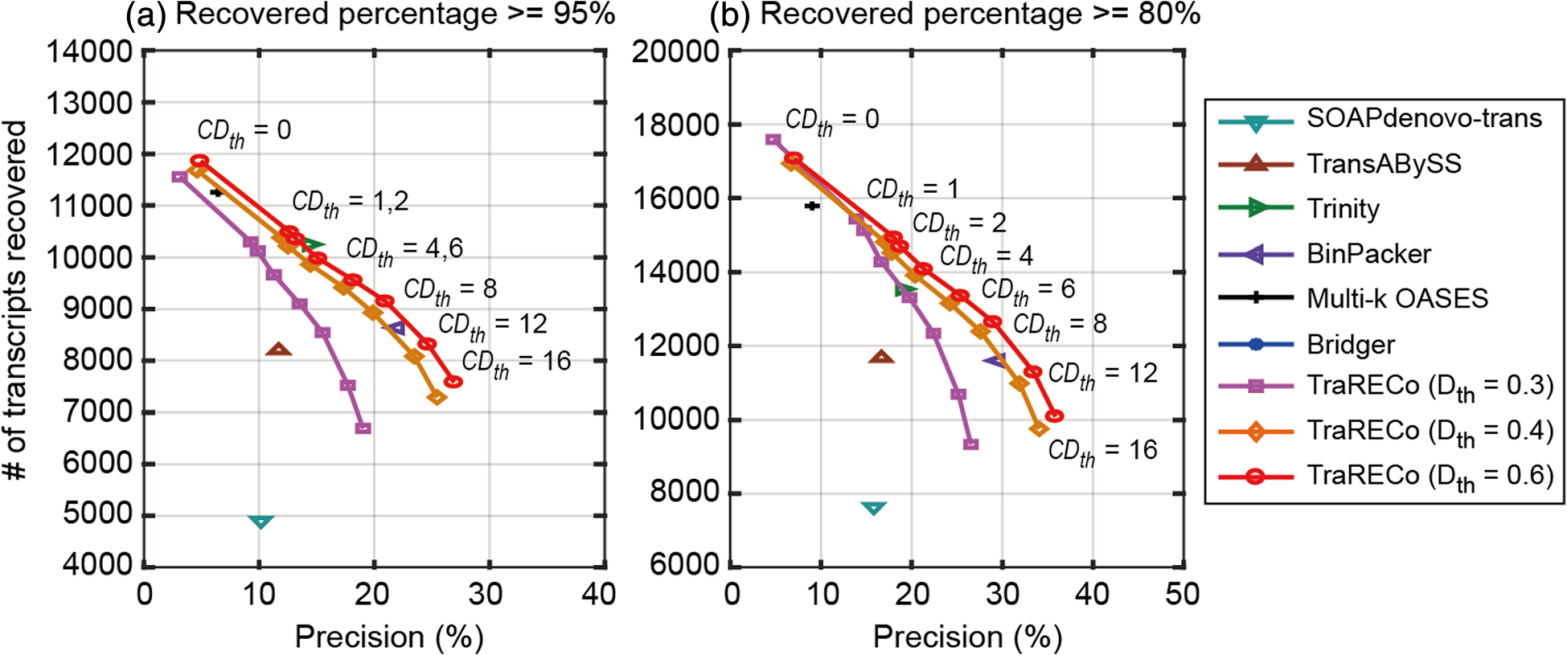
The number of transcripts recovered versus precision for the paired-end read data SRX062280 (Mouse sample). Target coverage = 95% (**a**) and 80% (**b**), respectively

The results are available in Table 1 and Fig. 1 for SRR445718, and Table 2 and Fig. 2 for SRX062280. Figures 1 and 2 show the precision versus the number of recovered transcripts for a target coverage (recovered percentage) of (a) 95% and (b) 80%. In the figures, the results for the assemblers other than TraRECo are shown as a single point, while the results for TraRECo are exhibited as a curve in which each point corresponds to a *CD*_*th*_ of 0, 1, 2, 4, 8, 12, and 16. Note that TraRECo jointly detects isoforms and estimates their abundance (expression level). *CD*_*th*_ is the abundance (coverage depth) cutoff at which we discarded those candidates with an abundance estimate below this value as most of these were highly likely to have been read error artifacts (*CD*_*th*_ = 0 means that we considered all of the paths obtained from the final splicing graph regardless of the abundance estimates). With various values of this cutoff, the performance of TraRECo was exhibited as a line for a given *D*_*th*_.

**Table 1:** A comparison of the number of transcripts found and matched to a reference for the single-end reads SRR445718 (Human sample)

Assembler	# of candidates found	# of transcripts matched for Target coverage of		
		≥ 95%	≥ 90%	≥ 80%
SOAPdenovo-trans	48,462	2613	3522	5349
Trans-ABySS	87,686	3626	4781	6967
Trinity	82,865	4008	5279	7680
BinPacker	20,612	3486	4314	5685
Multi-k OASES	202,152	5120	6737	9520
Bridger	58,217	4013	5093	7017
TraRECo, *D*_*th*_ = 0.06, *CD*_*th*_ = 0	219,402	5366	7010	10,168
TraRECo, *D*_*th*_ = 0.06, *CD*_*th*_ = 1	118,342	4845	6308	9078
TraRECo, *D*_*th*_ = 0.06, *CD*_*th*_ = 2	109,551	4774	6209	8901
TraRECo, *D*_*th*_ = 0.06, *CD*_*th*_ = 4	79,073	4537	5890	8393
TraRECo, *D*_*th*_ = 0.06, *CD*_*th*_ = 6	58,396	4217	5436	7642
TraRECo, *D*_*th*_ = 0.06, *CD*_*th*_ = 8	47,095	3937	5026	6973
TraRECo, *D*_*th*_ = 0.06, *CD*_*th*_ = 12	35,506	3396	4305	5880
TraRECo, *D*_*th*_ = 0.06, *CD*_*th*_ = 16	28,997	2959	3749	5089

Except for Trinity, Cutadapt was used for read data trimming

Target coverage = 95%, 90% and 80%. For TraRECo, the results with *D*_*th*_ = 0.06 and *CD*_*th*_ = 0, 1, 2, 4, 6, 8, 12 and 16 are shown

**Table 2:** A comparison of the number of transcripts found and matched to a reference for the paired-end read data SRX062280 (Mouse sample)

Assembler	# of candidates found	# of transcripts matched for Target coverage of		
		≥ 95%	≥ 90%	≥ 80%
SOAPdenovo-trans	48,234	4902	6088	7634
Trans-ABySS	70,052	8202	9673	11,680
Trinity	71,415	10,247	11,645	13,543
BinPacker	34,838	8979	10,151	11,685
Multi-k OASES	174,724	11,256	13,224	15,789
TraRECo, *D*_*th*_ = 0.06, C*D*_*th*_ = 0	240,907	11,864	13,980	17,076
TraRECo, *D*_*th*_ = 0.06, C*D*_*th*_ = 1	82,522	10,480	12,355	14,927
TraRECo, *D*_*th*_ = 0.06, *CD*_*th*_ = 2	78,226	10,347	12,183	14,688
TraRECo, *D*_*th*_ = 0.06, *CD*_*th*_ = 4	65,823	9978	11,706	14,080
TraRECo, *D*_*th*_ = 0.06, *CD*_*th*_ = 6	52,465	9555	11,166	13,353
TraRECo, *D*_*th*_ = 0.06, *CD*_*th*_ = 8	43,550	9146	10,638	12,643
TraRECo, *D*_*th*_ = 0.06, *CD*_*th*_ = 12	33,704	8313	9614	11,282
TraRECo, *D*_*th*_ = 0.06, *CD*_*th*_ = 16	28,132	7577	8708	10,087

Except for Trinity, the cutadapt SW was used for read data trimming

Target coverage = 95%, 90% and 80%. For TraRECo, the results with *D*_*th*_ = 0.06 and *CD*_*th*_ = 0, 1, 2, 4, 6, 8, 12 and 16 are shown

To show the impact of distance threshold, we ran TraRECo with *D*_*th*_ = 0.04, 0.05, and 0.06 for the human sample and 0.03, 0.04, and 0.06 for the mouse one, although Table 1 only contains the results for *D*_*th*_ = 0.06. The results in Figs. 1 and 2 indicate the impact of *D*_th_ on the performance of TraRECo. One can observe the improvement in both precision and sensitivity with a larger distance threshold up to *D*_*th*_ = 0.06, after which no further improvement in sensitivity was observed although the precision was slightly improved. From the performance curves for TraRECo, one can clearly see the performance improvement with larger values of *D*_*th*_ (up to 0.06). Note that with larger values of *D*_*th*_, more erroneous reads are made usable for the graph construction, improving the quality of coverage depth information used in the subsequent path search step, and eventually, the assembler performance. Our results suggest that *D*_*th*_ = 0.06 is a reasonable choice for obtaining good findings with real data, although it must be chosen carefully according to the error statistics, as suggested by our results for simulated reads reported later on.

#### Sensitivity versus precision

In Fig. 1 for the human sample, TraRECo demonstrated better precision than most of the assemblers, the exception being BinPacker. When considering only the number of transcripts recovered, TraRECo with *D*_*th*_ = 0 and multi-k OASES attained the best results; for the number of transcripts for the 95% and 80% targets, this method found more than 5000 and 9500, respectively, while most of the other assemblers found only around 4000 or fewer and 7700 or fewer, respectively. Of course, such high sensitivity in TraRECo with *D*_*th*_ = 0 and multi-*k* OASES might have been obtained only at the cost of precision. For the mouse sample (shown in Fig. 2), when compared at the same precision or at the same sensitivity, TraRECo showed slightly better results than most of the other assemblers, the exceptions being Trinity and BinPacker. Moreover, we could not obtain any results for Bridger due to runtime errors that could not be corrected.

#### Transcript length mismatch

**Fig. 3:**
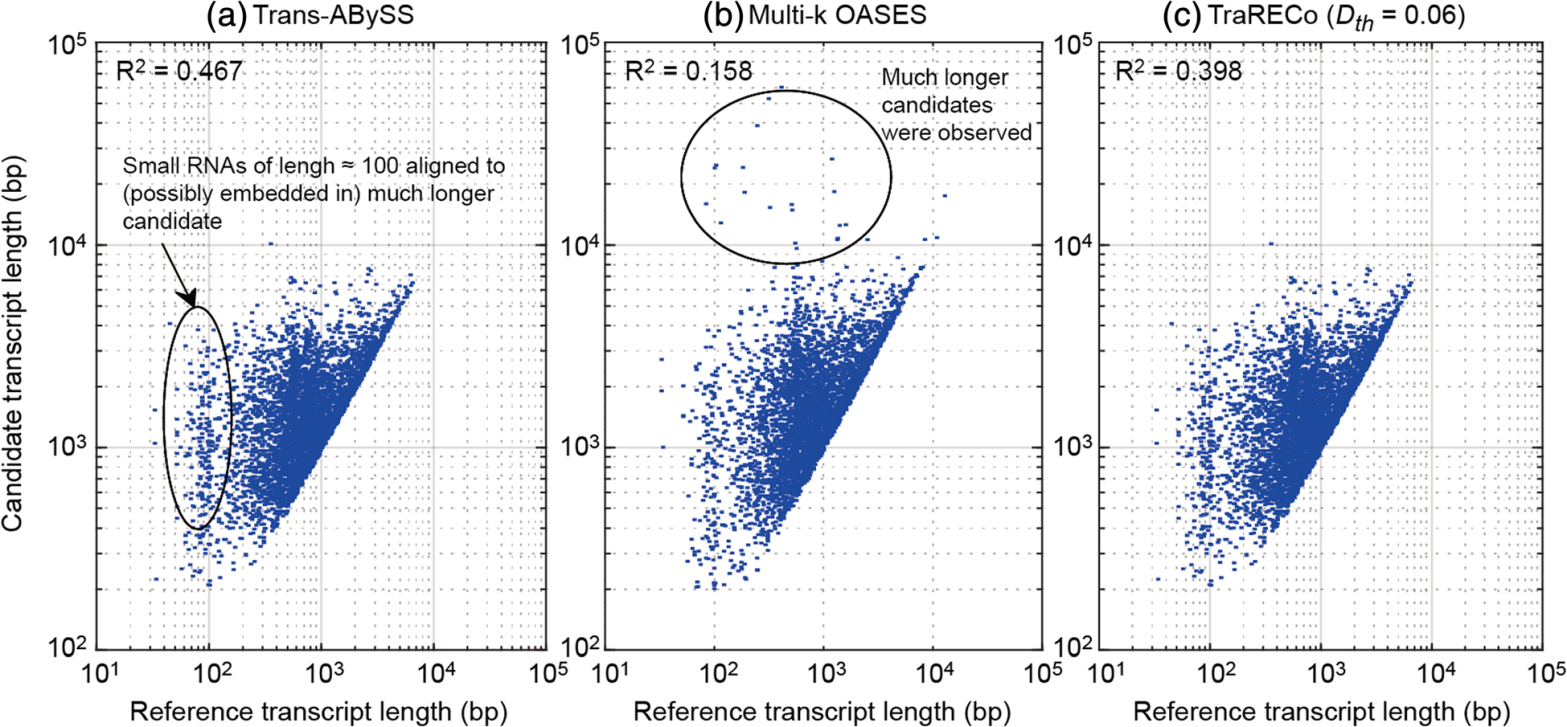
Scatter plot representing length correlation between reference and candidate transcripts matched with 95 or higher coverage of reference for human sample (SRR445718). **a** Trans-ABySS, (**b**) multi-*k* OASES, (**c**) TraRECo (*D*_*th*_ = 0.06)

**Fig. 4:**
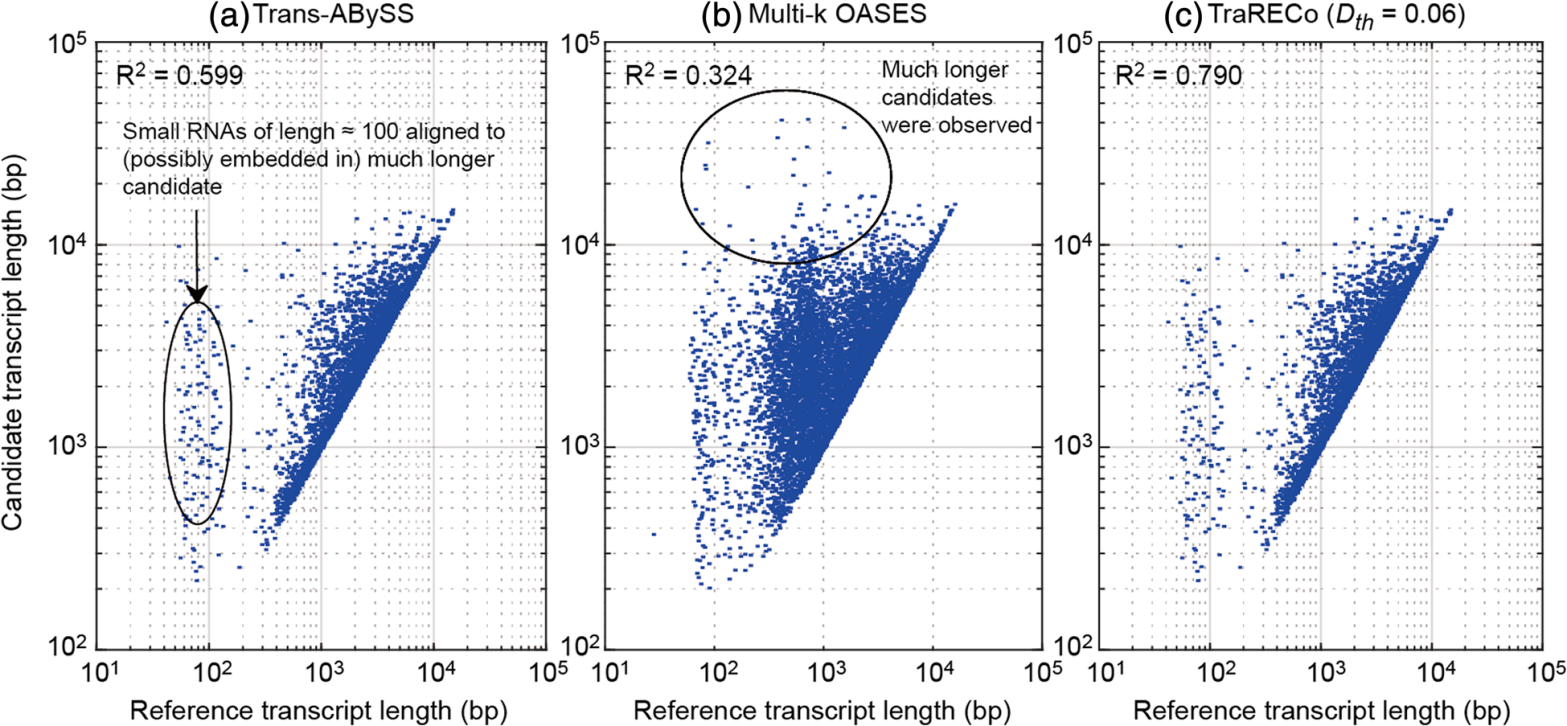
Scatter plot representing length correlation between reference and candidate transcripts matched with 95 or higher coverage of reference for mouse sample (SRX062280). **a** Trans-ABySS, (**b**) multi-*k* OASES, (**c**) TraRECo (*D*_*th*_ = 0.06)

In the sensitivity measures obtained here, we allowed only one reference transcript (with the highest alignment length) to be paired with each candidate. However, when comparing the length of candidate transcripts with those of their paired reference, one can see that there were big differences between the two. Figures 3 and 4 show scatter plots of the candidate (assembled) transcript lengths and their paired references for SRR445718 and SRX062280, in which we show only those for Trans-ABySS, multi-k OASES, and TraRECo (*D*_*th*_ = 0.06, *CD*_*th*_ = 4). For the human sample, the R^2^ measurements for these were 0.467 (best), 0.158 (worst), and 0.398, respectively, and for the mouse sample, 0.6, 0.324 (worst) and 0.79 (best), respectively. For other assemblers, the R^2^ measurements for human (mouse) were 0.382 (0.593), 0.408 (0.511). 0.351 (0.44), and 0.349 (not available) for SOAPdenovo-trans, Trinity, BinPacker, and Bridger, respectively. In fact, there were noticeable mismatches in all of the assemblers and we believe that the mismatch between the two lengths does not necessarily mean a worse performance since they simply stem from artificial gene fusion due to sequence repeats.

#### Wide sense (WS) sensitivity

Figures 3 and 4 show that many reference transcripts, including small RNA sequences with lengths of around 100 bp, were aligned to a much longer candidate transcript, indicating that each candidate might represent multiple reference transcripts due to artificial gene fusion. Perhaps these small RNA sequences recovered with the de novo assemblers were not expressed at all, while detected because other larger transcripts contained them as a part of their entire sequence. Once we consider such gene fusion and the mismatch as a general phenomenon (even though a good assembler should be able to combat sequence repeats in an efficient way), it will be interesting to check how many (and which) reference transcripts were aligned to a single candidate. Considering such fusion as a usual occurrence, we defined the WS sensitivity as the number of reference transcripts that are recovered by any one of candidates for a given minimum target coverage, i.e. by allowing multiple references paired with one candidate.

**Fig. 5:**
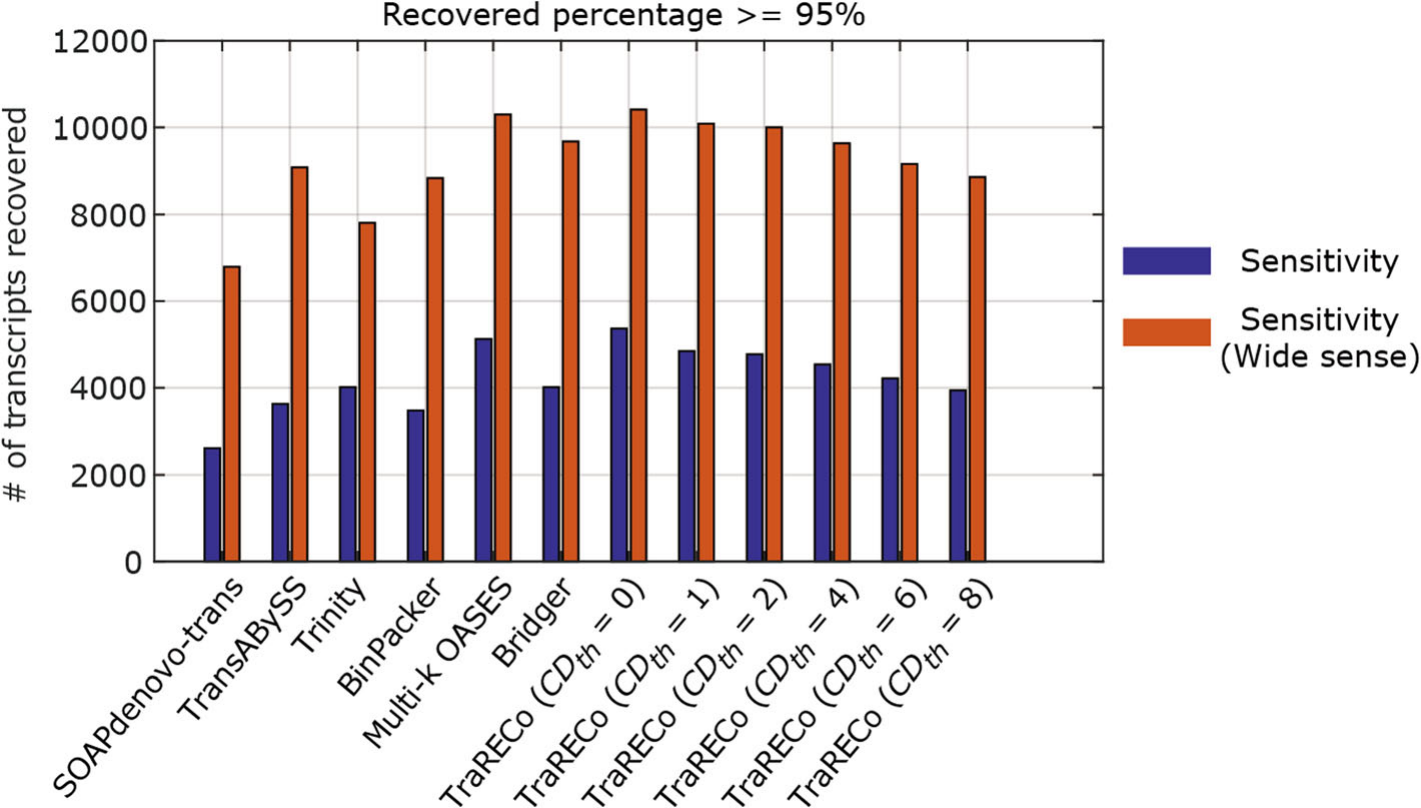
A comparison of sensitivities with wide sense sensitivities for human sample (SRR445718). Target coverage ≥ 95%

**Fig. 6:**
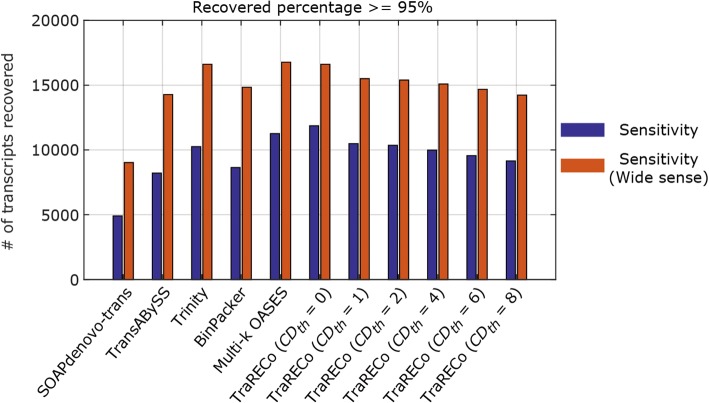
A comparison of sensitivities with wide sense sensitivities for human sample (SRX062280). Target coverage ≥ 95%

Figures 5 and 6 compare the WS sensitivity (the number of references recovered by ‘any’ candidate) with the sensitivity (allowing only one candidate for each reference). As shown, there are noticeable differences between the sensitivity and WS sensitivity for all of the assemblers. Specifically for the human sample, the WS sensitivities were twice as high as the sensitivities for all of the assemblers considered, the largest percentage difference being that of Bridger. The overall WS sensitivity looked to have a similar pattern to the assemblers considered herein, although Bridger and Trinity attained quite good performances for the human and mouse samples when considering both WS sensitivity and precisions simultaneously. One thing to note here is that when considering WS sensitivity, one may need to redefine precision since many candidate transcripts (isoforms) found with each assembler share the same exons and it might be more appropriate to use, for example, the number of nucleotides in the splicing graphs, which were not available for most of the assemblers.

### Results for simulated reads

We were able to perform a more in-depth investigation of assembler performance (including abundance estimation) using simulated reads as we had prior knowledge of the exact set of isoforms and their expression level. This will be interesting even though the simulated reads might have had different characteristics from the real ones and the performance demonstrated for the simulated reads could have been different from the practical performance with real data.

#### Data generation and prior knowledge

The simulated data we used were generated with the Flux Simulator [23] using the UCSC mm9 reference genome and its annotation. The Flux Simulator first randomly generated expression levels for all of the transcripts in the annotation and then simulated the library preparation (including reverse transcription, fragmentation, and size selection) to obtain reads through the sequencing process. Using the default error models, we generated 41 M reads of length 100 bp. In addition to the reads, the Flux Simulator also provided the following additional information for each isoform generated, which was used for the in-depth investigation provided in this section.

1. Expressed coverage (covered fraction): the expressed coverage is the percentage of an isoform’s length that is covered by the generated reads.
2. Sequenced number: the number of reads sequenced for a given transcript such that the coverage depth (expression level per base) can be obtained as the number of sequenced reads times the read length divided by the transcript length times the expressed coverage.

#### Parameter settings and pre/post processing

Through a similar procedure as for real reads, we compared TraRECo with the other assemblers previously used. We used the same parameters for all of the assemblers except for a *k*-mer length of 31 for Bridger and BinPacker (the suggested value for the mouse sample as in [12]). Using the candidate isoforms obtained from each assembler, we ran BLASTN to attain how many reference transcripts were matched to the candidates. Here, we did not use mm9 Ensembl transcriptome and instead, created a reference transcriptome by using the gene annotation and the expression level profile obtained from the supplemental files generated along with the simulated reads. The reference transcriptome contained the exact set of transcripts from which the simulated reads were generated.

#### Sensitivity versus precision

**Fig. 7:**
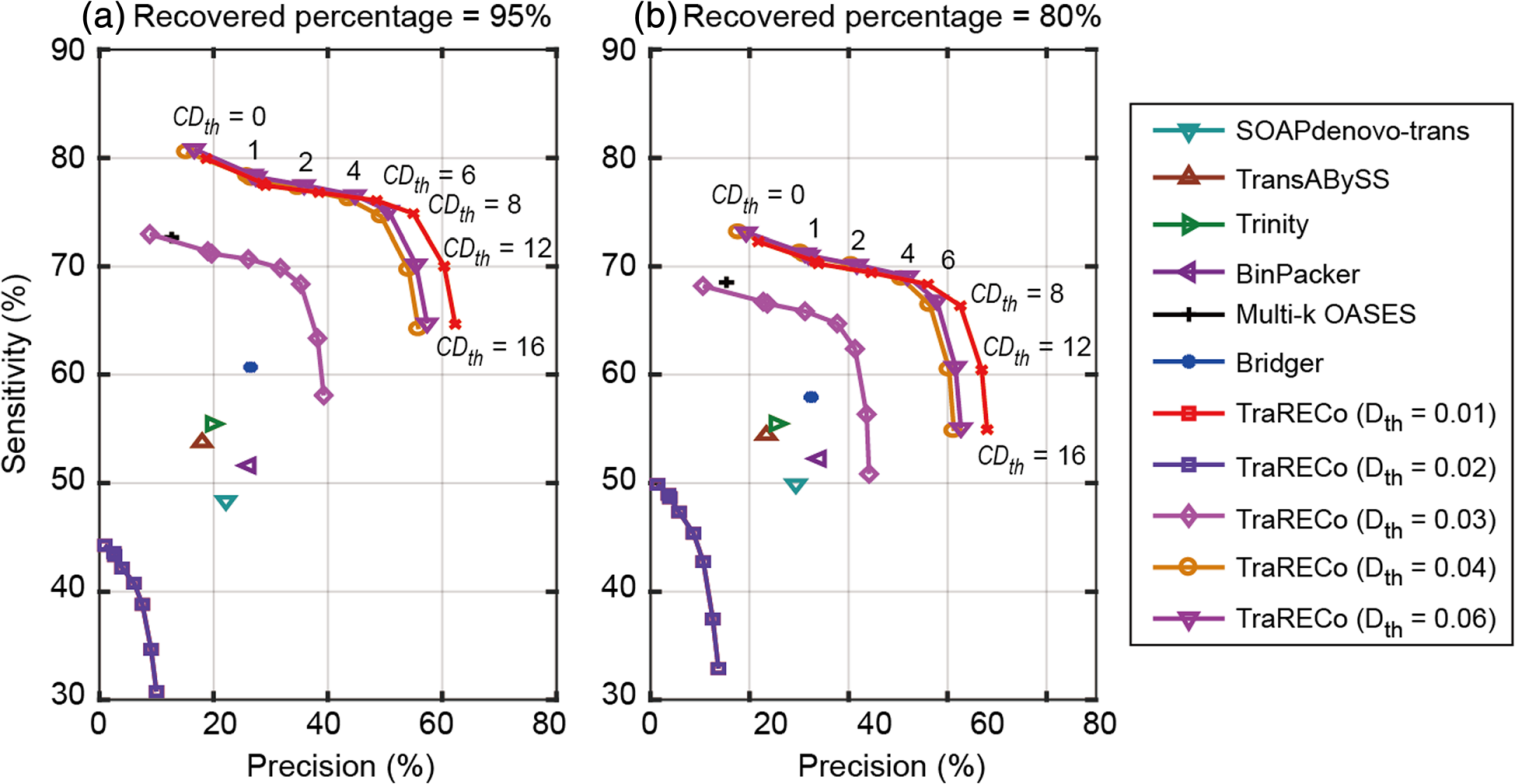
Sensitivity versus precision for the single-end simulated reads. Target coverage = 95% (**a**) and 80% (**b**), respectively. Sensitivity is defined as the number of recovered transcripts divided by the number of reference transcripts with its expressed coverage larger than the target coverage (provided in the last row of Table 3)

Table 3 contains the number of transcript candidates found with each assembler and the number of recovered references with a coverage greater than or equal to the specified target value (95%, 90%, or 80%). In the last row, we also report the number of transcripts with an expressed coverage greater than or equal to the specified target value. As a matter of fact, the number of recovered transcripts for a given target coverage could not exceed this number for the same coverage values. The sensitivity was defined as the percentage of the recovered transcripts among all of the reference transcripts with an expressed coverage greater than or equal to the target value. The sensitivity versus precision for the simulated reads is shown in Fig. 7, in which compared with the other assemblers, TraRECo showed the best performance for both sensitivity and precision. Differently from the real reads, the performance of TraRECo with the simulated reads compared to the other assemblers was considerably better. However, this does not necessarily indicate its better performance with real data since the characteristics of simulated reads can be different from those of real reads.

**Table 3:** A comparison of the number of transcripts found and matched to a reference for the simulated reads (Mouse)

Assembler	# of candidates found	# of transcripts matched for Target coverage of		
		≥ 95%	≥ 90%	≥ 80%
SOAPdenovo-trans	21,640	4792	5730	6353
Trans-ABySS	29,642	5330	6280	6934
Trinity	27,962	5499	6314	7067
BinPacker	19,489	5117	5920	6655
Muli-k OASES	56,885	7201	8132	8727
Bridger	22,737	6015	6781	7378
TraRECo, *D*_*th*_ = 0.05, *CD*_*th*_ = 0	42,275	7923	8739	9205
TraRECo, *D*_*th*_ = 0.05, *CD*_*th*_ = 1	27,005	7700	8511	8975
TraRECo, *D*_*th*_ = 0.05, *CD*_*th*_ = 2	26,398	7677	8481	8946
TraRECo, *D*_*th*_ = 0.05, *CD*_*th*_ = 4	19,813	7613	8395	8840
TraRECo, *D*_*th*_ = 0.05, *CD*_*th*_ = 6	15,540	7540	8298	8704
TraRECo, *D*_*th*_ = 0.05, *CD*_*th*_ = 8	13,502	7420	8110	8451
TraRECo, *D*_*th*_ = 0.05, *CD*_*th*_ = 12	11,503	6941	7473	7696
TraRECo, *D*_*th*_ = 0.05, *CD*_*th*_ = 16	10,290	6408	6821	6997
The number of Transcripts generated with its expressed coverage ≥ Target coverage		9911	11,538	12,733

Target coverage = 95%, 90% and 80%. For TraRECo, the results with *D*_*th*_ = 0.05 and *CD*_*th*_ = 0, 1, 2, 4, 6, 8, 12 and 16 are shown

#### Transcript length mismatch and WS sensitivity

**Fig. 8:**
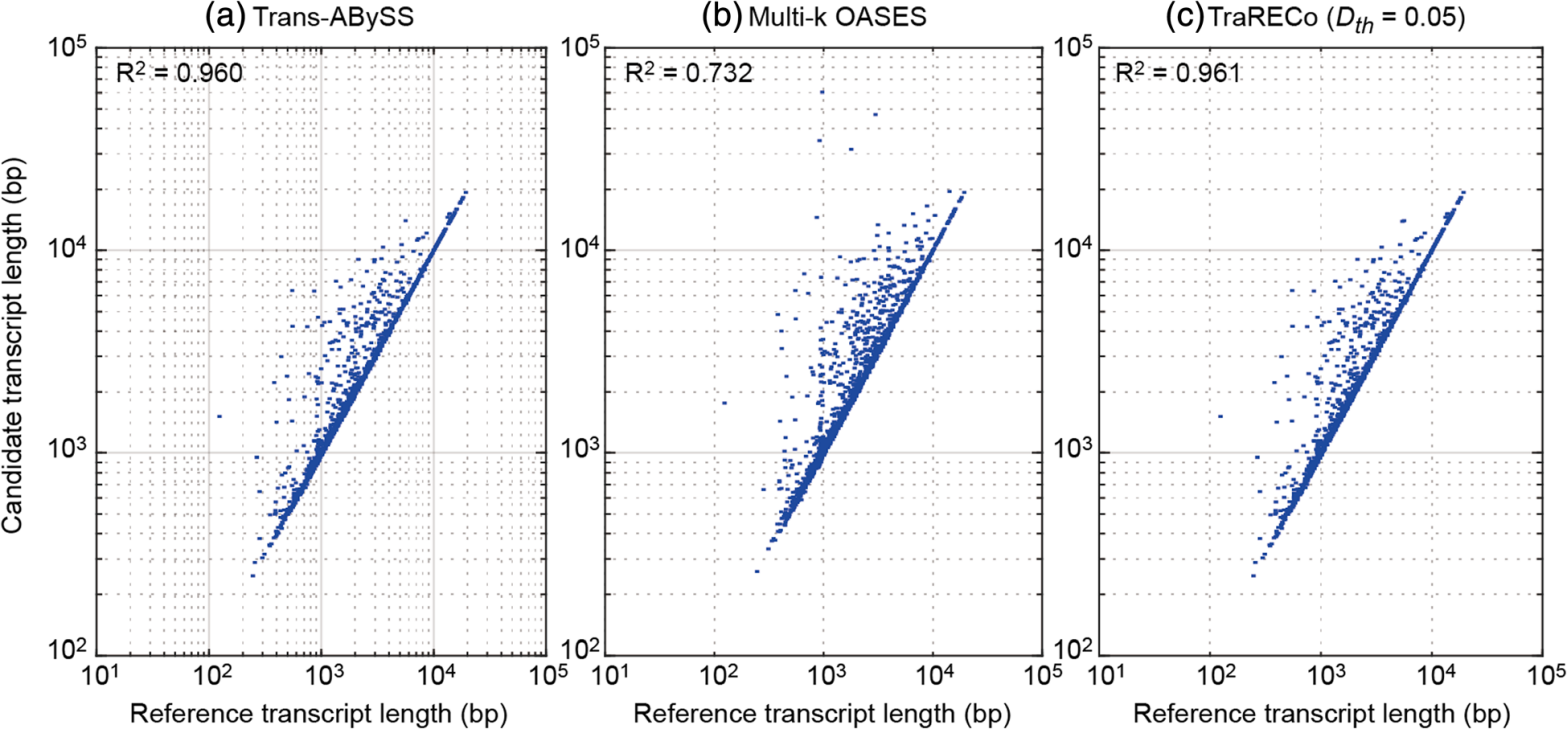
Scatter plot representing length correlation between reference and candidate transcripts matched with 95% or higher coverage of reference for the simulated read. **a** Trans-ABySS, (**b**) multi-*k* OASES, (**c**) TraRECo (*D*_*th*_ = 0.05)

**Fig. 9:**
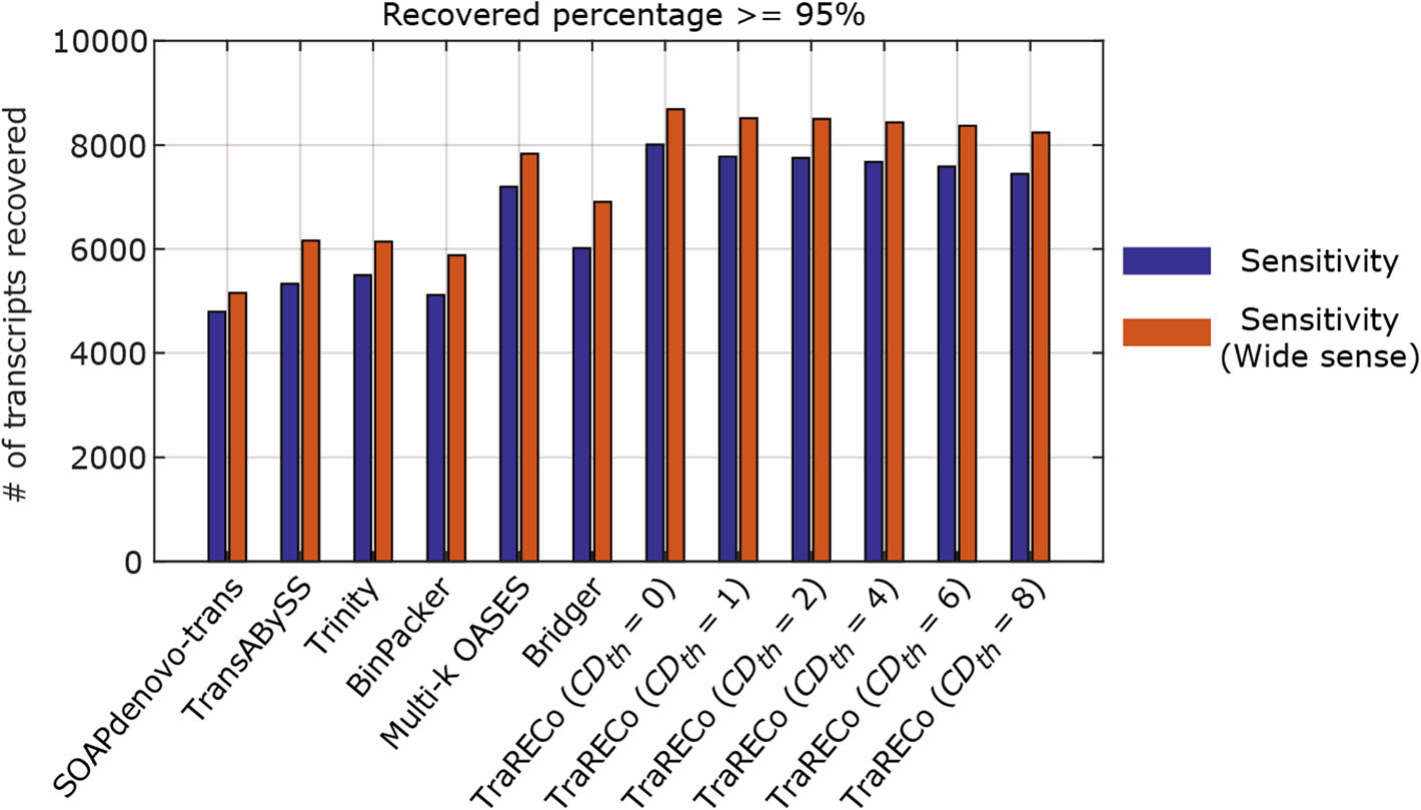
A comparison of sensitivities with wide sense sensitivities for simulated reads. Target coverage ≥ 95%

As with the real data, we checked length mismatch and WS sensitivity for the simulated data, as shown in Figs. 8 and 9. In Fig. 8, we showed only those for Trans-ABySS, multi-*k* OASES, and TraRECo (*D*_*th*_ = 0.05, *CD*_*th*_ = 4). Compared with those for the real data, the results with the simulated reads showed much better matches between the candidate and the reference transcripts and almost no small RNA sequences were detected. The R^2^measurements for SOAPdenovo-trans, Trans-ABySS, Trinity, BinPacker, multi-*k* OASES, Bridger, and TraRECo (*D*_*th*_ = 5, *CD*_*th*_ = 4) were 0.990 (best), 0.962, 0.960, 0.894, 0.732 (worst), 0.944, and 0.961, respectively. Figure 9 shows a comparison of sensitivity with WS sensitivity. As can be inferred from Fig. 8, there was no considerable difference between sensitivity and WS sensitivity for the assemblers.

#### Abundance and sensitivity

**Fig. 10:**
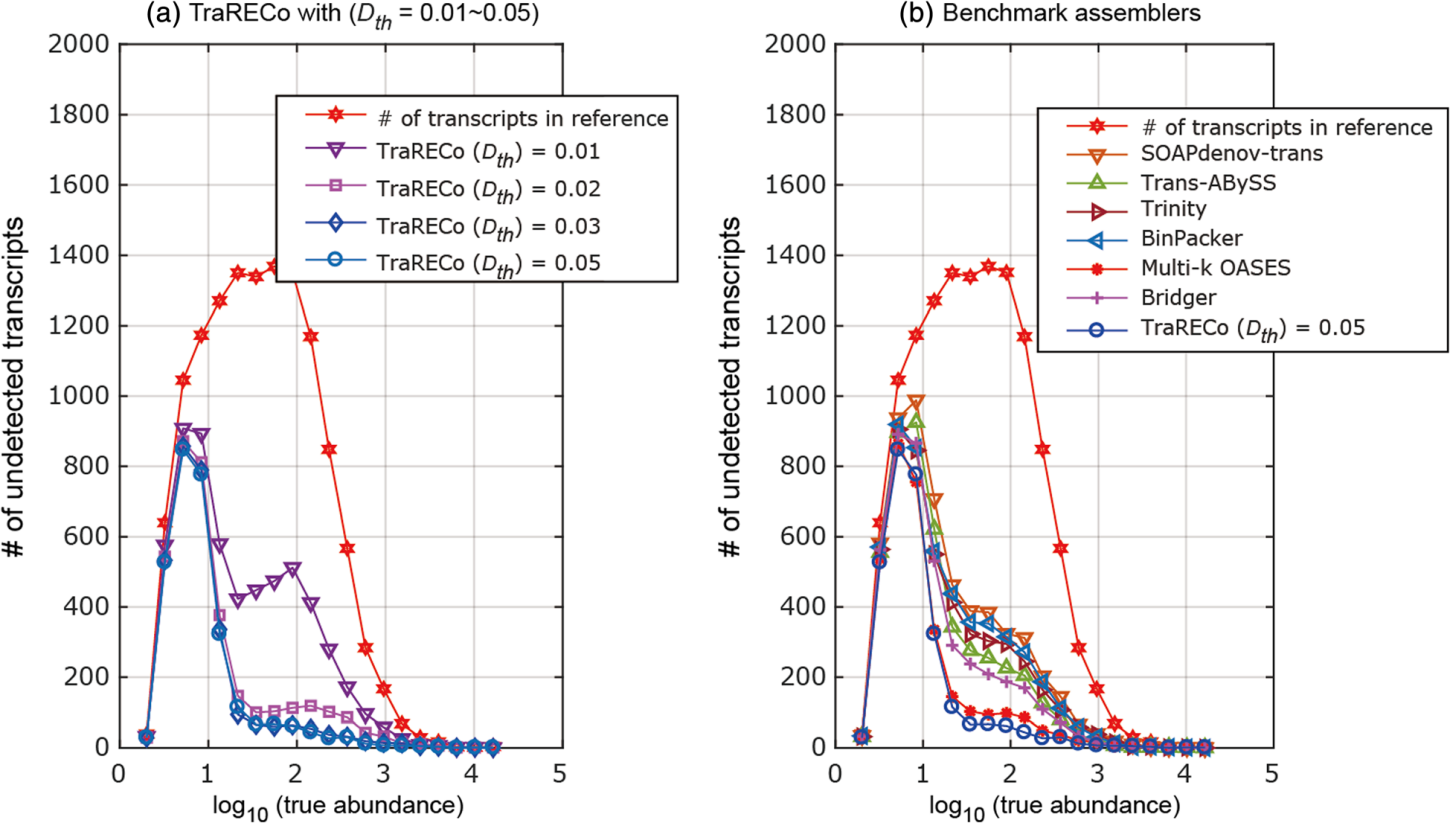
Histogram of the number of undetected transcripts (in wide sense) for simulated reads. Target coverage ≥ 80%. True abundance is shown in log-scale. To give insight into the percentage of undetected transcript, the number of all reference transcripts were also shown

**Fig. 11:**
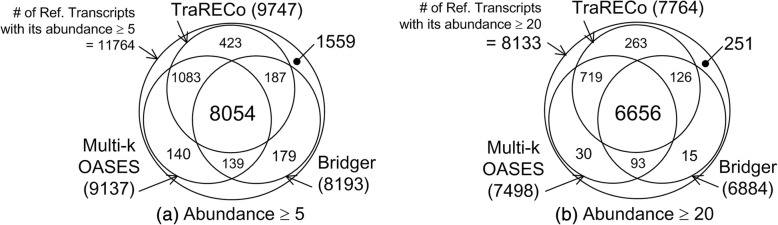
Venn diagram showing the number of transcripts detected that are common among various assemblers for the simulated reads. Target coverage 80%. Considered only those reference transcripts with its abundance being equal to or greater than (**a**) 5 and (**b**) 20

Figure 10 show the number of undetected transcripts with their true abundances approximately equal to the abscissas, where to obtain an insight into the percentage of undetected transcripts, the number of reference transcripts is also shown. We used 30 bins for true abundance from 0 to 4.2 on a log-scale. Figure 10a shows only TraRECo with *D*_*th*_ = 0.01, 0.02, 0.03, and 0.05, which clearly shows an improvement as *D*_*th*_ increased. Although our expectation was for some improvement, especially for the low-level expressed transcripts, the results show a marginal improvement for low-level expressed transcripts but considerable improvement for transcripts with medium expression levels (from 20 to several hundred on a normal scale (1.3~ 2.5 on a log-scale)). When compared with the other assemblers, TraRECo showed a slight improvement for transcripts with a low expression level. There are two things to note: (1) most of the assemblers, including TraRECo, could not successfully detect those transcripts with an abundance of fewer than 10 (1 on a log-scale); and (2) even with medium to high expression levels, non-negligible portions of transcripts could not be detected. This is true even when we consider sensitivity as it is slightly lower than the WS sensitivity, as shown in Fig. 9. Figure 11 shows the number of recovered transcripts common among TraRECo, multi-*k* OASES, and Bridger for a target coverage of 80% or higher (we selected these three assemblers as they showed the highest sensitivity). We can see that (1) a considerable percentage of candidates (68~ 81%) were commonly processed and (2) a non-negligible percentage (1559/11,764 ≈ 13% for abundance ≥ 5 and 251/8133 ≈ 3% for abundance ≥ 20) could still not be recovered.

#### Abundance estimation performance

**Fig. 12:**
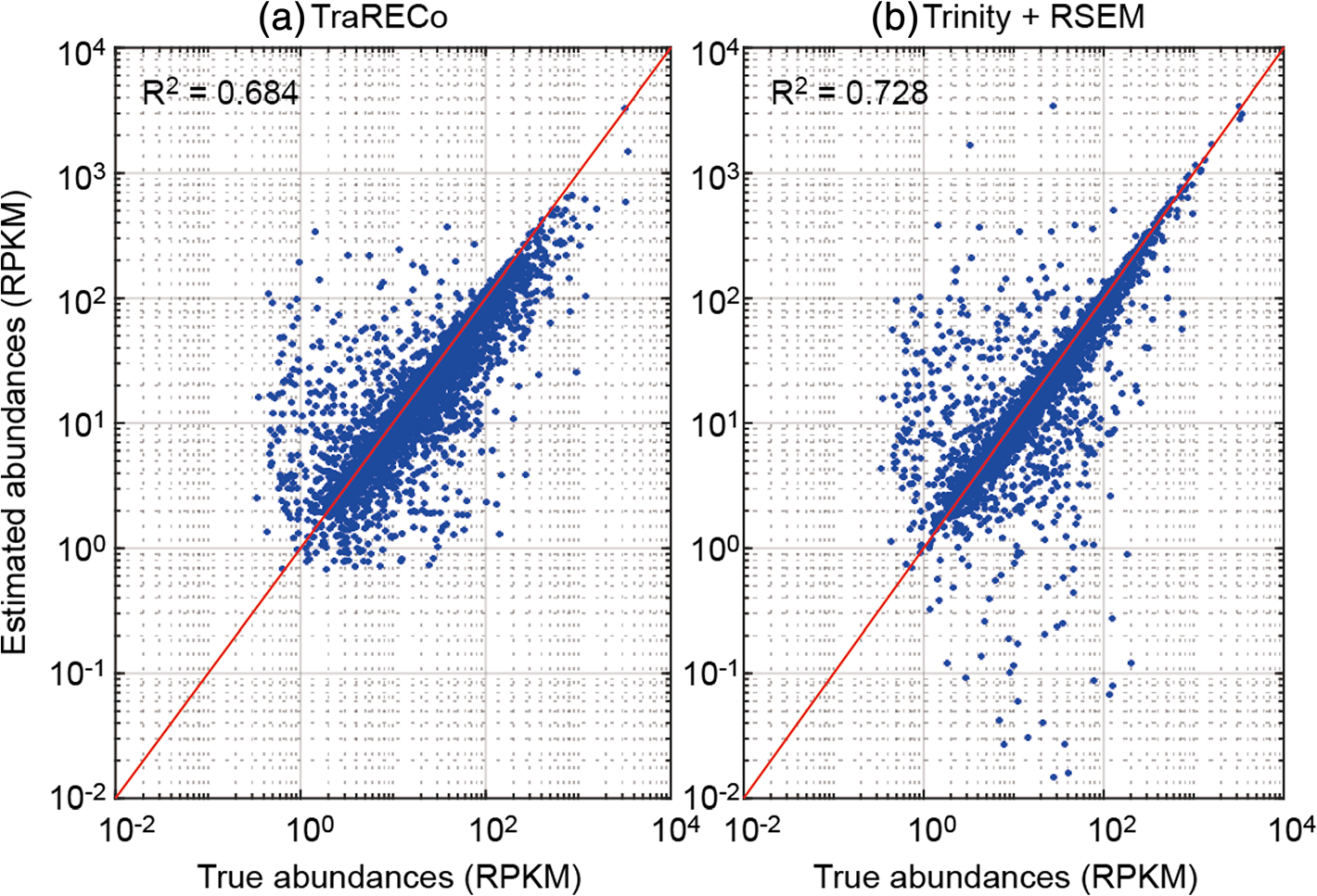
Abundance estimation accuracy of TraRECo (**a**) and Trinity + RSEM (**b**)

Note that TraRECo also provides abundance estimates for each candidate, with which we could trade sensitivity for precision by controlling the coverage depth threshold *CD*_*th*_. Although abundance estimation is a secondary issue in a de novo transcriptome assembler, it was of interest to see how accurate it was for TraRECo. Figure 12 shows a comparison of the abundance estimation performance between Trinity+RSEM and TraRECo. The latter produces abundance estimates for all of the detected isoforms along the assembly procedure, while the former provides a separate abundance estimation package called RSEM, with which estimates are obtained by realigning the reads to the assembled isoforms. RSEM provides abundances in Transcripts Per Kilobase Million (TPM) and Fragments Per Kilobase Million (FPKM), while TraRECo provides it in Reads Per Kilobase Million (RPKM) and raw coverage depth. Hence, we used the effective transcript length and expected read count provided by RSEM to obtain the estimates in RPKM as 10^9^*n*_*k*_/*l*_*k*_*N* where *n*_*k*_ and *l*_*k*_ are the expected read count and effective length of the *k*^th^ transcript and *N* is the sum of *n*_*k*_’s for all *k*.

We selected those isoforms with a coverage of 80% or higher and compared their abundance estimates with the true abundances provided as prior knowledge. The R^2^ measurements for the TraRECo and Trinity+RSEM were 0.684 and 0.728, respectively. Although TraRECo attained less accurate abundance estimates than Trinity+RSEM, they were still quite close. Another thing to note is that TraRECo tended to slightly underestimate the abundances for a large portion of transcripts even though the big differences evident using Trinity+RSEM were seldom (Fig. 12a). The underestimation tendency was because some of the reads were discarded in the contig growing step of TraRECo, as they could not be aligned within the specified distance margin.

### Assembly quality measurements using DETONATE

For real data, we do not have the exact set of transcripts and the evaluation can only be based on the known transcripts disregarding the unknown (yet possibly existing) isoforms, while simulated data may have different characteristics from real data. Given that the ground truth is unknown for real data, DETONATE [24] or TransRate [25] can be used as a more reliable measure of de novo transcriptome assembly. To provide insight into how well the assembled candidates represent the data in an efficient way, we used DETONATE, which provides two types of assembly quality measure, namely RSEM-EVAL without and REF-EVAL with a reference transcriptome. In our experiments, we used the former, which is a measure of how well and efficiently the assembled transcripts represent the read data.

Table 4 contains the results with the RSEM-EVAL method. For the real human sample (SRR445718) and the simulated reads, the best RSEM-EVAL scores with TraRECo (*CD*_*th*_ = 2) were − 1,391,489,463 and − 1,480,014,038, respectively, while the best scores among the other assemblers were − 1,450,049,444 (Trans-ABySS) and − 1,482,875,599 (Trinity), respectively. For the real mouse sample (SRX062280), the best score with TraRECo (*CD*_*th*_ = 16) was − 11,282,292,195, while the best among the other assemblers was − 11,260,544,990 (SOAPdenovo-Trans). For the latter sample, it seems that the number of candidate transcripts was the dominant factor in achieving a better score as TraRECo with *CD*_*th*_ = 16 (having the least number of candidates) obtained the best among all of the TraRECo scores, which gradually worsened with smaller *CD*_*th*_ values.

**Table 4:** A comparison of DETONATE (RSEM-EVAL) scores for the three data samples

Assembler	SRR445718 (Human)	SRX062280 (Mouse)	Simulated reads
SOAPdenovo-trans	-1,714,875,885	−11,260,544,990 ^a^	−1,601,887,363
Trans-ABySS	− 1,450,049,444 ^a^	− 11,304,600,611	− 1,528,116,522
Trinity	− 1,489,443,203	− 11,327,881,219	− 1,482,875,599 ^a^
BinPacker	− 1,479,768,971	− 11,281,390,921	−1,620,059,120
Muli-k OASES	−2,333,599,533	−11,623,498,121	−2,031,443,441
Bridger	−1,479,768,971	Not available	−1,540,527,574
TraRECo, *CD*_*th*_ = 0	−1,604,604,900	−12,088,126,158	− 1559,358,185
TraRECo, *CD*_*th*_ = 1	−1,401,116,205	− 11,421,827,023	−1,481,790,008
TraRECo, *CD*_*th*_ = 2	−1,391,489,463 ^a^	− 11,402,670,499	−1,480,014,038 ^a^
TraRECo, *CD*_*th*_ = 4	−1,392,964,314	−11,372,430,684	− 1,486,956,413
TraRECo, *CD*_*th*_ = 6	− 1,419,050,587	− 11,347,202,193	−1,506,415,171
TraRECo, *CD*_*th*_ = 8	−1,452,606,806	− 11,327,444,550	−1,530,794,253
TraRECo, *CD*_*th*_ = 12	−1,529,402,883	− 11,300,573,327	−1,581,922,133
TraRECo, *CD*_*th*_ = 16	−1,598,153,693	− 11,282,292,195 ^a^	−1,630,265,385

Best performance among benchmark assemblers and among TraRECo with different coverage depth thresholds were marked with ^a^. For TraRECo, *D*_*th*_ = 0.05 was used for simulated read, while 0.06 for real reads

### Computational burden and runtime

Most of the existing assemblers took from 2 to 20 h except for multi-*k* OASES which ran for a few days to perform Velvet many times for the different *k*-mers and combined them to obtain the final splicing graph. TraRECo took around 64 h for the simulated reads and 150~ 180 h for the real reads, which were much longer than those with existing assemblers. There are two reasons for the much longer run time with TraRECo. First, it uses the direct alignment of the reads to the contigs, and in the worst case scenario, the computational complexity of the full alignment test grows according to (*NL*)^2^, where *N* is the number of reads and *L* is the read length. This is a bottleneck for the runtime with TraRECo. One thing to note is that as the contig growing step compares the reads with the contigs rather than with other reads, the complexity grows as *NL*⋅*N*_*c*_*L*_*c*_, where *N*_*c*_ is the number of contigs and *L*_*c*_ is the average contig length. Since *N*_*c*_*L*_*c*_ is typically much smaller than *NL*, the computational complexity is far less than what might be expected with square growth. A rough estimation of *N*_*c*_*L*_*c*_ is *NL* divided by the average coverage depth (abundance) which, as we saw in Fig. 8, is approximately 100. Another reason is that TraRECo was originally developed using MATLAB™, a proprietary software tool, while most of the assemblers were written using C/C++. Although C/C++ version of TraRECo is now available, the analysis provided here was performed using MATLAB™ version and the runtime was measured based on the latter. As MATLAB™ typically runs much more slowly than C/C++, we can save some proportion of the runtime by using C/C++, which is a software development issue rather than a bioinformatics one. Most of all, as our focus in this study was on the development of new methods rather than on the software used, the runtime was considered as a secondary issue.

## Discussion

Sensitivity and precision are two primary performance measures for de novo transcriptome assemblers and a tradeoff between the two criteria certainly exists. To maximize the performance, many de novo transcriptome assemblers perform assembly in two steps: (1) building a splicing graph and (2) searching for plausible paths. In the former step, a de Briujn graph approach has been widely adopted for use in most of the existing assemblers since its computational burden only increases linearly with the read data size. One problem with a de Bruijn graph is that it is not well suited to combat read errors and sequence repeats, and to overcome this problem, Schulz et al. [8] proposed a multi-*k* approach where de Bruijn graphs are constructed separately for many different *k*-mers. Although it takes a lot more iterations to obtain the final splicing graph, multi-*k* OASES provides the highest sensitivity among all of the existing assemblers for all of the samples including simulated reads. However, multi-*k* OASES has been shown to be the worst in terms of precision among all of the assemblers, while the best is the recently proposed BinPacker, especially for human samples. In between these two extreme cases, Bridger and Trinity are a good compromise in terms of the two performance criteria.

Compared to these de Bruijn graph approaches, TraRECo provides a new framework for de novo transcriptome assembly by combining the consensus matrix-based error correction procedure with direct read alignment based on the greedy approach. Through the work presented in this study, we confirmed that the proposed contig growing procedure using consensus matrices could combat read errors efficiently in that the sensitivity of TraRECo with low coverage depth threshold (*CD*_*th*_) was even better than multi-*k* OASES. As mentioned before, this improvement was achieved by making additional reads having errors usable in the graph construction step. Without an error-aware alignment test, many of the reads with errors will eventually be discarded, resulting in a poor splicing graph. However, utilizing the consensus matrix based read alignment we could make these reads usable to improve the coverage depth information used in the subsequent path search steps and finally the assembler performance. This aspect is certainly different from the simple read-error removal or error correction based on the topological structure of the de Bruijn graph approach and the difference could make the proposed approach an alternative method, at least for the splicing graph construction step, even though it has a higher computational burden due to direct alignment test.

On the other hand, the direct alignment of reads to build the contigs made us able to resort full connection information of short reads to suppress the impact of sequence repeats of a length less than the minimal overlap width used for read alignment. Furthermore, the improvements over a broad range of abundances shown in Fig. 10 for the simulated reads support this argument. Although there is still a problem in that any transcripts with low expression level could not be connected during the contig growing step, we could alleviate the problem by connecting the contigs with a smaller connection threshold (*C*_*th*_) before performing the junction search and the graph construction step. Resorting to the full connection information of a read played an important role in the junction search and the group (graph) decomposition step as well, by which we could utilize the junction overlap width for the group/graph decomposition to facilitate the subsequent joint isoform detection and abundance estimation.

For the final step, we borrowed the idea of IsoLasso to jointly detect isoforms and estimate their abundance. IsoLasso is a simple yet quite powerful method for the subsequent path search step, even though there are approaches that are more sophisticated such as Bridger and BinPacker. However, our approach was different from IsoLasso’s in that if there are loops in the graph, then we allow a segment to be included in a path more than once. The original IsoLasso did not take this into account simply because loops never occur in reference-based assembly as these techniques utilize known gene annotation, but they can appear in de novo assembly as an artifact of a sequence repeat.

The results from the simulated reads also show that many challenges still remain unsolved, especially for the path search step. Specifically, a non-negligible portion of the transcripts with their expression level being high enough (a read depth greater than 20) could still not be recovered by the existing de novo assemblers, including TraRECo, which appears to have been mainly due to sequence repeats by which many transcripts/isoforms were merged together. To solve such problems, one may need to devise more sophisticated methods to decouple merged transcripts/isoforms and artificial gene fusion.

## Conclusions

Many existing de novo transcriptome assemblers are based on the de Bruijn graph approach, which builds a splicing graph in linear time of the data size but suffers from read errors that make the splicing graph complicated. Because of this, it seems natural that recent approaches such as Bridger and BinPacker have focused more on a reliable path search to improve the precision. Another research direction has been forged by Schulz et al. [8] to suppress the impact of read errors and short repeats by using the multiple *k*-mers approach. The study presented here pursued the same objective as this and we believe it was successful in the sense that the proposed approach showed the highest sensitivity when precision was not considered. TraRECo also attained a good performance even when comparing both sensitivity and precision at the same time. Although the computational burden of direct read alignment can be much higher than the single *k*-mer de Bruijn graph approach, it seems that its computational burden was far less than the worst squared complexity due to its recursive computation providing an alternative to de novo assembly. Overall, TraRECo was able to provide reliable splicing graph construction, which is an important issue since de novo assembly is mainly to explore as yet undiscovered isoforms and must be able to represent as many reads as possible in an efficient way.

## Implementation

**Fig. 13:**
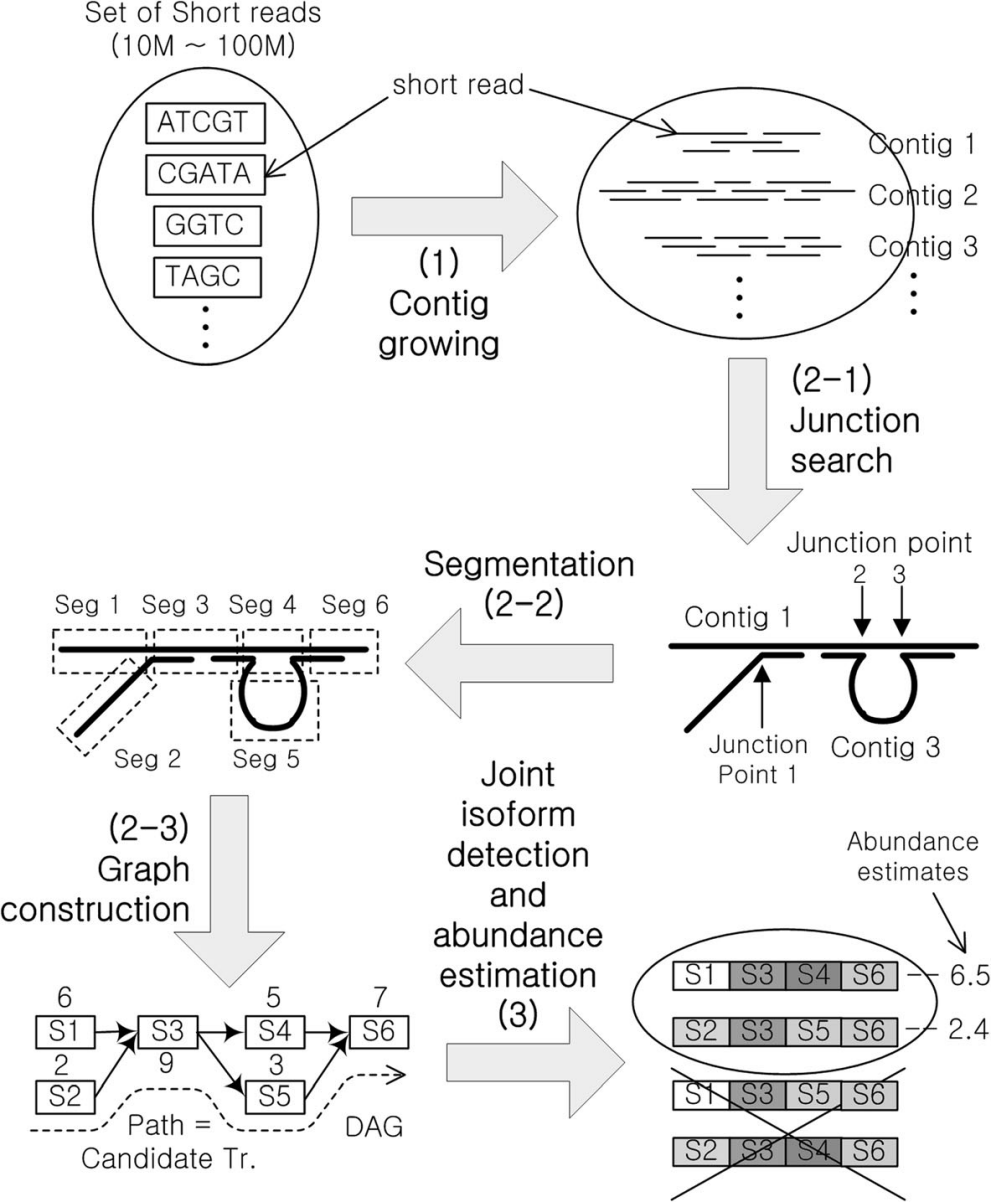
The entire workflow of TraRECo assembly procedure consisting of (1) contig growing, (2) junction search and splice graph construction and (3) joint isoform detection and abundance estimation. In contig growing step, we employed greedy approach and consensus-matrix based error-aware alignment test. In junction search, we test alignment of prefix and suffix of a contig with other contigs. The aligned prefix and suffix contain chimeric reads that span over two consecutive exon, even though they might have been aligned because of sequence repeat. The inner end of aligned prefix or suffix will possibly be the junction boundary between two consecutive exons. In joint isoform detection and abundance estimation, we used a modified version of IsoLasso, by which one can remove the transcript candidates according to their estimated abundance

The entire procedure for the proposed assembly consists of three parts: (1) contig growing, (2) junction search and graph construction, and (3) joint isoform detection and abundance estimation, as summarized in Fig. 13. The contig growing step utilizes the greedy approach that has been widely adopted for DNA assembly, e.g. Zhang et al. [26], SSAKE [27], SHARCGS [28], and VCAKE [29], as it is more suitable for error correction and one can utilize the full connection information of a read. The second step is to search for junctions among the contigs and then to construct the graph, which consists of nodes (representing a segment of the base sequence) and edges (representing the connections between the segments). In this step, the read coverage for each base is tracked to obtain the coverage depth profile for each contig and segment. Finally, this information is used to jointly detect isoforms and estimate abundances. This procedure differs from previously reported methods as follows:

1. In the contig growing step, we use a “consensus” matrix that holds an alignment profile represented by the base count for each location of a contig. This profile makes it possible to identify errors and check whether any similar sequences have been merged into a single contig.
2. This alignment profile is also tracked in the subsequent junction search and the graph construction step delivered to the final stage where one can jointly detect isoforms and estimate abundances.

Throughout this section, we provide details of the assembly process and highlight the key features of the proposed assembler.

### Contig growing based on a consensus matrix

**Fig. 14:**
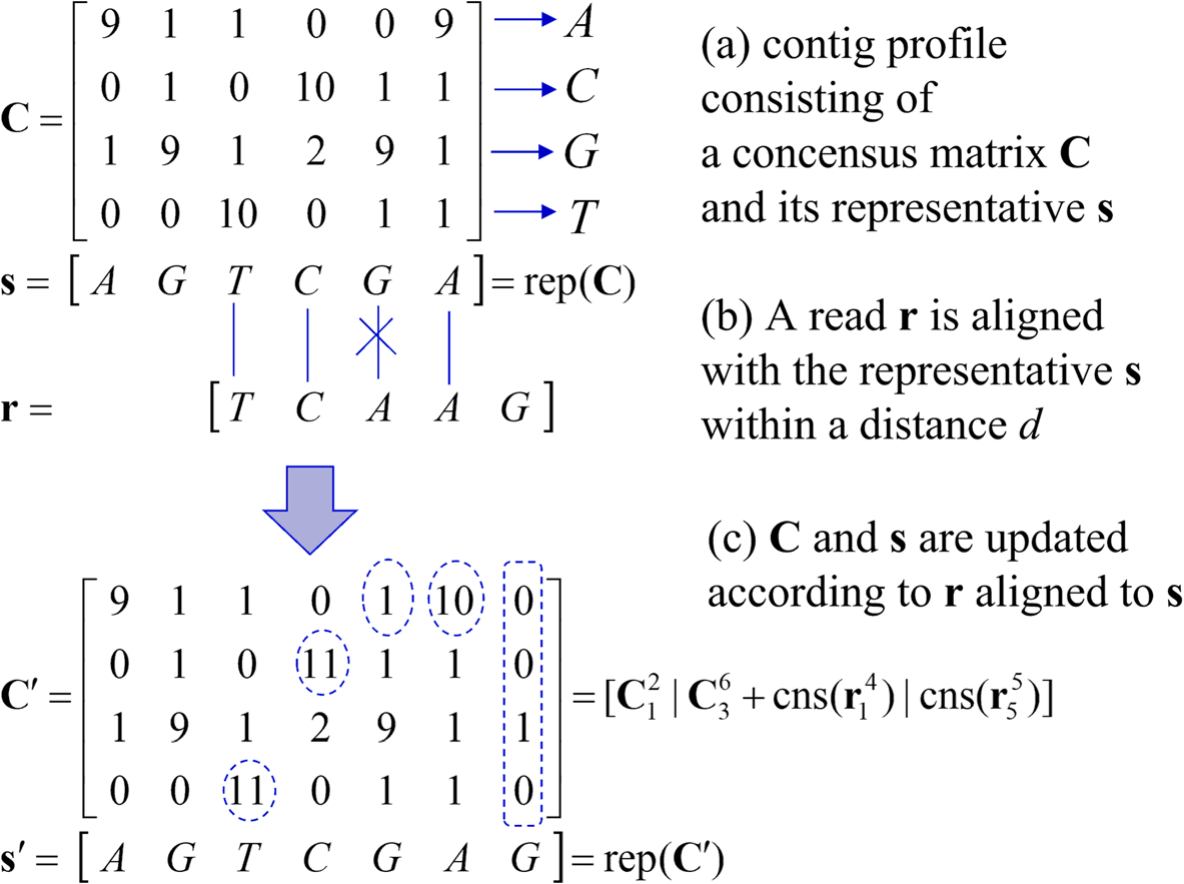
An example of contig profile update when a read ***r*** of length 5 partially overlaps with ***s*** = rep(***C***) of length 6 from the right (differing only one base among 4). The contig profile ***C*** is extended to and replaced with the updated contig profile ***C***′, which is now of length 7. The updated elements are marked with dashed circle

The contig grower builds contigs by aligning short reads to all of the contigs in the contig pool and extending the one with the highest match in a recursive manner. We use the name to emphasize the recursive nature of the algorithm, which alleviates the squared complexity with data size, and it proceeds as follows. Let Ψ be the set of contigs found so far and Ξ be the set of short reads. We select a read from Ξ and try to align it with all of the contigs in Ψ. Next, we choose the contig with the widest overlap for the selected read. If the read completely overlaps with the contig, we merge the former with the latter or extend the latter if the former only partially overlaps it. If there is no contig having an overlap longer than or equal to a predefined value, we simply add the read to Ψ as a new contig. This procedure is repeated until all of the reads in Ξ have been processed. One key feature in our contig growing is that we use a consensus matrix to provide read error correction, as summarized in Fig. 14.

In the proposed scheme, a contig of length *l* is represented by a consensus matrix ***C*** of size 4 × *l*, and the corresponding representative vector ***s*** of length *l*. Each row in ***C*** corresponds to a base in {A, C, G, T} and each element in ***C*** is the base count of reads aligned to this contig. On the other hand, each element in ***s*** corresponds to the row index with the highest count at each column in ***C***, i.e. *s*_*i*_ = argmax_*i* ∈ {1, 2, 3, 4}_*c*_*i*, *j*_, where the row indices {1,2,3,4} correspond to the letters {A,C,G,T}.

The representative **s** is used to test the alignment with a read as follows. Let us consider an alignment test of read ***r*** of length *m* with a representative ***s*** of length *l*. Without any loss of generality, we assume *l* ≥ *m*. Three cases can occur: (1) complete overlap, (2) partial overlap from the left or right of the reference ***s***, and (3) no overlap. Let ${\boldsymbol{t}}_l^n$ be a portion of vector ***t*** such that ${\boldsymbol{t}}_l^n=\left[{t}_l,{t}_{l+1},\dots {t}_n\right]$ for *l* ≤ *n* and ${\boldsymbol{t}}_l^n=\left[\ \right]$, a null vector of length 0, if *l* > *n*. The same notation can also be applied to a matrix, i.e. ${\boldsymbol{T}}_l^n$ is a matrix consisting of the *l*^th^ to *n*^th^ columns of matrix ***T***. Subsequently, one can write $\boldsymbol{r}=\left[{\boldsymbol{r}}_1^n|{\boldsymbol{r}}_{n+1}^m\right]$ for integer 0 ≤ *n* ≤ *m*. Based on this notation, we say that ***r*** completely overlaps with ***s*** if for some integer *a* in [1, *l* − *m* + 1], ${d}_H\left({\boldsymbol{r}}_1^m,{\boldsymbol{s}}_a^{a+m-1}\right)\le m{D}_{th}$ with *m* ≥ *O*_*th*_, where *d*_*H*_(***a***, ***b***) is the Hamming distance between the two vectors ***a*** and ***b***, *D*_*th*_ is the normalized distance threshold such that *mD*_*th*_ is the number of errors allowed, and *O*_*th*_ is the minimum overlap width for an overlap to be considered as valid.

On the other hand, we say that ***r*** partially overlaps with ***s*** if *n* exists that satisfies one of the following conditions: (1) ${d}_H\left({\boldsymbol{r}}_{m-n+1}^m,{\boldsymbol{s}}_1^n\right)\le n{D}_{th}$ (partial overlap from the left) or (2) ${d}_H\left({\boldsymbol{r}}_1^n,{\boldsymbol{s}}_{l-n+1}^l\right)\le n{D}_{th}$ (partial overlap from the right) for *m* > *n* ≥ *O*_*th*_. If multiple *n*’s satisfy any of the conditions, we take the largest value. For notational simplicity, we define two functions: rep(⋅) and cns(⋅). The function rep(⋅) takes consensus matrix ***C*** and returns its representative sequence such that ***s =*** rep(***C***), while function cns(⋅) takes sequence ***s*** and returns a consensus matrix initialized by ***s***, i.e. each element of ***C =*** cns(***s***) is set to *c*_*i*, *j*_ = 1 if *i* = *s*_*j*_, or 0 otherwise. Now, we can describe the contig-profile update procedure using the two functions by considering 3 cases. Suppose that read ***r*** of length *m* partially overlaps with the representative ***s =*** rep(***C***) of length *l* ≥ *m* from the left/right of ***s*** and the overlap width obeys *m* ≥ *O*_*th*_, then we update the contig profile as follows:

1 $$ \mathrm{From}\kern0.17em \mathrm{the}\kern0.17em \mathrm{left}:\boldsymbol{C}\leftarrow \left[\mathrm{cns}\left({\boldsymbol{r}}_1^{m-n}\right)\left|{\boldsymbol{C}}_1^n+\mathrm{cns}\left({\boldsymbol{r}}_{m-n+1}^m\right)\left|{\boldsymbol{C}}_{n+1}^l\right.\right.\right], $$

2 $$ \mathrm{From}\kern0.17em \mathrm{the}\kern0.17em \mathrm{right}:\boldsymbol{C}\leftarrow \left[{\boldsymbol{C}}_1^{l-n}\left|{\boldsymbol{C}}_{l-n+1}^l+\mathrm{cns}\left({\boldsymbol{r}}_1^n\right)\left|\mathrm{cns}\left({\boldsymbol{r}}_{n+1}^m\right)\right.\right.\right]. $$

An example of a contig profile update for the partial overlap case is shown in Fig. 14. Suppose that read ***r*** of length *m* completely overlaps with the representative ***s =*** rep(***C***) of length *l* ≥ *m* at position *a*, then we update the contig profile accordingly:

3 $$ \mathrm{Complete}\ \mathrm{overlap}:\boldsymbol{C}\leftarrow \left[\left.{\boldsymbol{C}}_1^a\right|{\boldsymbol{C}}_{a+1}^{a+m}+\mathrm{cns}\left(\boldsymbol{r}\right)\left|{\boldsymbol{C}}_{a+m+1}^l\right.\right]. $$

If read ***r*** does not overlap with ***s*** or overlaps at a width less than *O*_*th*_, we simply add cns(***r***) to the contig pool as a new seed. The overall contig growing procedure is as follows:

Contig growing

*Input*: Ξ = {***r***_1_, ***r***_2_, …, ***r***_*N*_}

*Initialization*: Set seed contig, e.g., Ψ = {cns(***r***_1_)} and remove ***r***_1_ from Ξ.

*Loop*: For all ***r ∈*** Ξ

A. Test alignment of ***r*** with ***s =*** rep(***C***) for all ***C ∈*** Ψ

B. If there exist ***C*** having overlap width *n* larger than *O*_*th*_ (within distance *nD*_*th*_), select ***C*** having the widest overlap with ***r*** and update ***C*** using (1), (2) or (3) based on the overlap width and position

C. Otherwise, add ***r*** as new contig, i.e., Ψ ← Ψ + cns(***r***)

D. Remove ***r*** from Ξ.

*End loop*

*Output*: Ψ = {***C***_*k*_ : *k* = 1, 2, …}

#### Parameter settings

Two key parameters in the proposed contig growing procedure are the normalized distance threshold *D*_*th*_ and the overlap threshold *O*_*th*_. *D*_*th*_ is the normalized value per base such that *nD*_*th*_ is the maximally allowed number of different letters for a portion of a read of length *n* to be aligned with a contig representative, where *n* ≥ *O*_*th*_. If *D*_*th*_ is too small, reads with more errors will not be aligned with a target contig and may eventually be disregarded. On the other hand, if it is too large, many similar sequences from other isoforms might be merged together, resulting in artificial gene fusion. Such artifacts make the splicing graph complex and the subsequent joint isoform detection and abundance estimation complicated in exactly the same way as occurs the de Bruijn graph-based approach. However, in contrast to the de Bruijn graph-based approach, the artificial gene fusion from short repeats can be avoided by setting *O*_*th*_ to be relatively large (larger than the *k*-value in the de Bruijn graph-based approach) as long as the read length is much longer than a typical *k*. Moreover, note that it is undesirable to set *O*_*th*_ to be too large, especially when the read coverage is insufficient, i.e. for isoforms with a low expression levels. If one sets *O*_*th*_ to be large, true transcripts cannot eventually be connected in those regions where the read coverage is low. On the other hand, if one set it to be too small, the graph construction is vulnerable to short repeats, resulting in the possible merging of multiple isoforms with similar sequences. In fact, there is a tradeoff between the error correction capability and the complexity of the final splicing graph to be used for joint detection and abundance estimation and we need to be careful when setting the thresholds for *D*_*th*_ and *O*_*th*_.

#### Post contig combining and contig filtering

Post contig combining can be helpful, especially for those isoforms with a low expression level, and can be performed in the same way as contig growing but with a smaller overlap threshold, for which we defined the connection threshold denoted as *C*_*th*_. Although setting *C*_*th*_ smaller than *O*_*th*_ can result in undesired contigs becoming connected to each other due to sequence repeats, this can be identified at the junction search stage and eventually be resolved through the subsequent steps. One can also remove those nodes with a length of less than a certain threshold value and a read coverage depth of, say, less than 2 since they are highly likely to be short fragments that could not be aligned due to many errors.

### Junction search and graph construction

#### Junction search and contig grouping

In the second step of the procedure, we first search junctions by testing the alignment of the prefix and suffix of a contig with other contigs. As in reference-guided assembly, the junction between two or more exons can be identified by chimeric reads. That is to say, a contig having chimeric reads at its prefix or suffix will have a partial overlap in the middle of other contigs and, as shown in Fig. 13, the inner end of the aligned prefix or suffix will possibly be the junction point of two consecutive exons. Note that wrong junction points can be identified due to sequence repeats and we need to select carefully from among the many junction candidates (this issue is discussed shortly). The alignment test here is exactly the same procedure as the one in the contig growing step. Note that (1) the overlap width around a junction is smaller than the nominal read length as we are employing the greedy approach and (2) we need to carefully set the junction overlap threshold *J*_*th*_ since it plays the same role as the overlap threshold in the contig growing and filtering. Subsequently, based on the junction information collected for all of the pairs of contigs, one can group the contigs that are linked together, where each group (hopefully) represents a gene with multiple isoforms. In this work, we set *J*_*th*_ = 24.

#### Junction filtering and group decomposition

Sometimes, the group size appears to be very large, which corresponds to a complex graph when using a de Bruijn graph-based approach, and in this case, the isoform detection that follows become very complicated. At this stage, one can invalidate some of the junctions according to their confidence levels. In this approach, one can utilize not only the read coverage depth of the two contigs involved in each junction but also the overlap width and distance within the overlap region. In fact, it is quite risky to apply a fixed threshold to invalidate junctions because the overlap width around it is roughly proportional to the isoform expression level, which can be quite uneven among genes. Although more sophisticated junction filtering can be devised, we applied simple filtering as follows. For the given junction information of a group, we iteratively invalidate the junction with the smallest overlap width until the group size become less than or equal to a predefined number, say 40 (see [10] for the justification of a reasonable group size).

#### Segmentation and graph construction

Now, for each group of contigs, we segment the contigs (contig profiles) at each valid junction point (junction boundary) and build a segment connection matrix to finally construct the splicing graphs for each group of contigs having valid junctions with each other.

Splicing graph *G*(*N*,*E*) consisting of nodes *N* and edges *E* is a directed graph, possibly with loops due to sequence repeats longer than *J*_*th*_. In the splice graph, each node represents a segment having a segment profile (consensus matrix) and each edge connects one segment to another. The read coverage profile ***v*** of a segment with segment profile ***S*** of size 4 × *l* is obtained by summing all of the rows of ***S***, column by column.

#### Compacting the graph to make it minimal

We define indeg(*n*) and outdeg(*n*) as the number of input and output edge degrees, respectively, of node *n*, and src(*e*) and dest(*e*) as the source and destination of edge *e*, respectively. We say that two nodes *n* = src(*e*) and *n*′ = dest(*e*) are singly connected if and only if outdeg(*n*) = indeg(*n*′) = 1. Without any loss of information, one can combine these two nodes to make the graph minimal, in which none of the nodes is singly connected. By performing this, we assume later on that the splicing graph used for the joint isoform detection and abundance estimation is minimal.

### The joint isoform detection and abundance estimation

With splicing graph *G*(*N*,*E*) consisting of nodes *N* and edges *E*, one can now jointly detect isoforms and estimate their abundances based on the per-segment average coverage depth {*y*_*j*_: *j*∈*N*}. To this end, we tried IsoLasso [3], for which we slightly modified the procedures to make it fit into our framework.

Let Π be the set of all of the maximal paths starting from a node with input degree 0 and end at one with output degree 0. Typically, the number of all of the paths |Π| is larger than the number of true isoforms. The problem is to find the true isoforms only. At first, one can resort to paired-end reads to filter out those paths that are not compatible with any of the paired-end reads used in the contig growing step. However, even with such a filter, the problem still remains, i.e. there exist many branches caused by read errors which are all compatible with the reads as they come from the same set of reads. One can use IsoLasso [3], which was proposed to ease the problem of an under-determined system, to select a plausible set of candidate isoforms while taking into account cases where there are too many paths. IsoLasso is a constrained minimum mean-squared-error estimator that can be concisely written as a quadratic optimization problem:

4 $$ {\min}_{\boldsymbol{x},\lambda}\parallel \boldsymbol{y}-\boldsymbol{Ax}{\parallel}^2+\boldsymbol{\lambda} \parallel \boldsymbol{x}{\parallel}_1\kern1em \mathrm{subject}\kern0.34em \mathrm{to}\kern1em \boldsymbol{x}\succeq 0 $$

where ***x*** is a |Π|×1 vector in which each element is the abundance of the candidate isoform being estimated; ***y*** is an |*N*| |×1 vector in which the *i*^th^ element is the average read coverage depth of the *i*^th^ node (segment) obtained from the corresponding segment profile; and ***A =*** [*a*_*i*, *j*_] is an |*N*|×|Π| matrix with *a*_*i*, *j*_ being a non-negative integer, representing the number of inclusions of node *i* in path *j*. The constraint ***x*** ≽ 0 means that all of the elements of ***x*** must be non-negative. Although the original IsoLasso employed various additional constraints, we did not considered them here to make the problem simple.

Once we obtain ***x***, we take those paths (candidate isoforms) with a length larger than length threshold *L*_*th*_ and an estimated abundance larger than coverage depth threshold *CD*_th_. IsoLasso is a good option, especially when |Π| ≫; |*N*|, in which case one can reduce the support set by increasing parameter *λ*. The final step is to discard those candidates with a length shorter than length threshold *L*_*th*_ and an abundance less than coverage depth threshold *CD*_*th*_. A typical value of *L*_*th*_ is 200 if we do not take the short non-coding RNA sequences into account. On the other hand, setting *CD*_*th*_ needs to be carried out with care. With a high *CD*_*th*_, one can obtain a better precision although true isoforms with a low expression level may be removed, thereby degrading sensitivity, and vice versa. Although a better joint detector/estimator can be designed that further improves the performance, we use this rather simple estimator as our focus is on the proposed contig growing and graph construction scheme.

#### Dealing with loopy graphs

As mentioned, each element of ***A***, *a*_*i*, *j*_, can be larger than 1, which means that we allow a node to be included more than once to resolve loopy graphs caused by relatively long sequence repeats. Although it seldom occurs, we allow a node in a loopy graph to be included twice, i.e. by assuming that a sequence repeat will occurred only once, we discard those paths that have any nodes included more than twice or more than two nodes included more than once.

## Availability and requirements

Project name: TraRECo

Project home page: https://sourceforge.net/projects/trareco/

Operating system: Windows/Linux

Programming language: MATLAB and C/C++

License: GPL 3.0

## Abbreviations

FPKM: Fragments Per Kilobase Million
NCBI: National Center for Biotechnology Information
OLC: Overlap-layout-consensus
RPKM: Reads Per Kilobase Million
SRA: Sequence Read Archive
TPM: Transcripts Per Kilobase Million
WS: Wide sense
